# Parental Post-Traumatic Stress Symptoms as Predictors of Psychosocial Problems in Children Treated for Cancer

**DOI:** 10.3390/ijerph13080812

**Published:** 2016-08-11

**Authors:** Ryoko Nakajima-Yamaguchi, Nobuaki Morita, Tomohei Nakao, Takashi Shimizu, Yasukazu Ogai, Hideto Takahashi, Tamaki Saito, Yoji Nakatani, Takashi Fukushima

**Affiliations:** 1 Departments of Child Health, Faculty of Medicine, University of Tsukuba, 1-1-1 Tennodai, Tsukuba, Ibaraki 305-8575, Japan; t.shimizu@iseharahp.com (T.S.); tksfksm@md.tsukuba.ac.jp (T.F.); 2 Departments of Social Psychiatry and Mental Health, Faculty of Medicine, University of Tsukuba, 1-1-1 Tennodai, Tsukuba, Ibaraki 305-8575, Japan; nobuakim@nifty.com (N.M.); ogai.ys@md.tsukuba.ac.jp (Y.O.); hhd02063@nifty.ne.jp (T.S.); 3 Department of Radiation Oncology, University of Tsukuba Hospital, Amakubo 2-1-1, Tsukuba, Ibaraki 305-8576, Japan; tnakao@umin.ac.jp; 4 Department of Pediatrics, University of Tsukuba Hospital, Amakubo 2-1-1, Tsukuba, Ibaraki 305-8576, Japan; 5 Department of Pediatrics, Isehara Kyodo Hospital, 345 Tanaka, Isehara, Kanagawa 259-1187, Japan; 6 Office of Information Management and Statistics, Radiation Medical Science Center for the Fukushima Health Management Survey, Faculty of Medicine, Fukushima Medical University, 1 Hikarigaoka, Fukushima 960-1295, Japan; htaka@fmu.ac.jp; 7 Kubota Clinic, 3-2-4 Yokokawa, Sumida, Tokyo 130-0003, Japan; yojinaka47@yahoo.co.jp

**Keywords:** childhood cancer, child behavior, adolescent behavior, parent-child relations, communication, posttraumatic stress symptoms

## Abstract

The purpose of this study was to explore the association between psychosocial functioning of children treated for cancer and that of their parents. Factors associated with psychosocial functioning were also examined. The present study was a cross-sectional survey of 33 mothers and one father (mean age: 37.9), each of whom had a child that had been treated for cancer. The participants answered a package of questionnaires consisting of the Impact of Event Scale-Revised (IES-R), the Parent Experience of Child Illness (PECI), and the Child Behavior Checklist (CBCL). Information about the children’s illnesses was collected from medical records. The CBCL total problems *T* score was correlated with the parental IES-R total scores. Intensity of treatment independently predicted the variance of parental long-term uncertainty. In conclusion, psychosocial problems of children with cancer were associated with parental post-traumatic stress symptoms (PTSS). Provision of early, adequate support to parents who are vulnerable to PTSS will help not only the parents, but also their children with cancer.

## 1. Introduction

Survival rates for childhood cancer diagnosed before the age of 18 years have improved due to aggressive multidisciplinary therapeutic approaches. With the improvement of survival rates, much attention has been focused on the psychosocial functioning of children with cancer.

Most research into psychosocial functioning in children with cancer has shown that symptoms of depression and anxiety decreased over time [1,2,3,4]. There is also some evidence that behavioral difficulties in these children were slightly increased compared to healthy children [5]. On the other hand, research into psychosocial functioning in adults surviving childhood cancer revealed that certain groups among them rarely obtain advanced education, get a job, or get married, compared to the normal population [6]. These inconsistent results indicate that there can be prolonged psychosocial difficulties from surviving childhood cancer that cannot be evaluated sufficiently by a self-reported questionnaire regarding depression and anxiety. More of the multi-faceted aspects of the psychosocial functioning of children with cancer should be evaluated.

Previous research on the psychosocial functioning of parents of children with cancer also revealed inconsistent results. Parental emotional difficulties, such as depression and anxiety, have been reported to decrease after cancer treatment [7,8]. However, many parents continue to experience chronic post-traumatic stress (PTS) for a long time after their child’s treatment [9]. PTS symptoms (PTSS) consist of a continuum of key symptoms of PTS disease (PTSD). Assessment of PTSS has proven to be more broadly applicable to the treatment of children with cancer and their parents than formally confirming the PTSD diagnosis is [10]. Parents also reported persistent feelings of loss, uncertainty, and anxiety about the recurrence of the disease or the emergence of late effects in their child. Such illness-specific distress of the parents should be assessed in order to fully understand their psychosocial functioning [11,12].

Parental psychosocial functioning has been found to be positively correlated with psychosocial functioning in children [13,14]. Parents of children with cancer are the most important emotional resources for the child, and parents who have severe distress and PTSS may well have difficulty caring for their children. Children with cancer whose parents have severe distress and PTSS may receive insufficient emotional support and have difficulties coping with their situation. In addition, previous studies revealed that support from peers is important for adolescents with cancer [15].

The psychosocial functioning of the child with cancer, illness-specific distress, and PTSS of the parents will be influenced by demographic and medical factors, such as the child’s age at diagnosis or the type of treatment. Many previous studies have shown that objective medical factors, such as the severity of disease and the intensity of treatment, are not associated with the psychosocial functioning of children with cancer. However, many of those studies excluded children who received relatively intensive treatment, such as for brain tumors [16,17]. The comparison of the severity or intensity of treatment of childhood cancer is difficult because these cancers are a heterogeneous mix.

Compared to western countries, the trend of parents making a full disclosure of the child’s diagnosis has progressed slowly in Japan [18,19]. However, even in cultures in which full disclosure is a common practice, parents often find it difficult to tell their child about the disease [20,21]. In western countries, the benefits of truthful disclosure have been examined [8,21,22,23], but there have been few such studies in Japan [24].

The purposes of this study were threefold. The first was to determine the proportions of children with cancer having a range of clinically relevant emotional and behavioral problems and the proportion of their parents having PTSS. The second was to clarify whether there were any associations between the children’s behavioral functioning and their parents’ psychosocial functioning. The third was to explore demographic, medical, and social factors associated with these children’s behavioral functioning and with their parents’ psychosocial functioning.

We hypothesized that parental distress and PTSS are associated with the emotional and behavioral functioning of children with cancer. In addition, we hypothesized that the intensity of treatment and social factors will be associated with parental distress, PTSS and the emotional and behavioral functioning of children with cancer.

## 2. Methods

### 2.1. Participants

Children of parents who were potential participants were identified from a list of children who were diagnosed and treated for childhood cancer between 1997 and 2007 at a single pediatrics department of a university hospital in Japan. Eligibility for inclusion were the following: (1) children aged four to 18 years at the time of enrollment; (2) children who had completed hospital treatment and were disease-free at time of enrollment; and (3) children who had scheduled follow-up appointments at the hospital between March and September 2008. The parents who accompanied their children were invited to participate at the outpatient ward.

### 2.2. Assessments

#### 2.2.1. Child Behavior Checklist (CBCL/4-18)

The four- to 18-year-old version of the Child Behavior Checklist (CBCL/4-18) is a parent-reported measure for children aged four to 18 years concerning the past six months [25]. It consists of eight subscales: Withdrawn (e.g., withdrawn, does not get involved with others), Somatic Complaints (e.g., overtired without good reason), Anxious/Depressed (e.g., unhappy, sad, or depressed), Social Problems (e.g., acts too young for his/her age), Thought Problems (e.g., cannot get his/her mind off certain thoughts), Attention Problems (e.g., cannot concentrate, cannot pay attention for long), Delinquent Behavior (e.g., does not seem to feel guilty after misbehaving), and Aggressive Behavior (e.g., destroys his/her own things). These eight subscales comprise two composite scales (Internalizing and Externalizing problems). The *T* scores of the Internalizing, Externalizing, and Total Problems subscales were standardized values based on the percentile scores obtained from investigation of the normal Japanese population [26]. A *T* score of 59 (84th percentile) or lower was classified as Normal, a *T* score between 60 and 63 (85th–90th percentile) was classified as Borderline Clinical, and a *T* score of 64 (91st percentile) or higher was classified as Clinical Range. Internal consistency values for the Externalizing, Internalizing, and Total Problems scores in this study were 0.96, 0.87, and 0.96, respectively.

The questionnaire also asks respondents to indicate the number of their child’s close friends, with the use of four check boxes, which indicated “none”, “1”, “2” or “3” and “4” or “more”. Each respondent is asked to check one box.

#### 2.2.2. The Impact of Event Scale—Revised (IES-R)

The IES-R is a 22-item self-report instrument that assesses three symptoms of PTSD: intrusion (e.g., any reminder brings back feelings about the event), avoidance (e.g., I stay away from reminders about it), and hyper-arousal (e.g., I felt irritable and angry) [27]. High scores indicate a high frequency of PTSS. The Japanese version of the IES-R has good internal consistency, and test-retest reliability has been reported [28]. We modified the instrument, as suggested by the authors, to identify PTSD symptoms in a cancer population by replacing the generic trauma of the original scale with the specific trauma of the child’s cancer and its treatment. The internal consistency value for the Japanese version of the IES-R total scores in this study was 0.87.

#### 2.2.3. Parent Experience of Child Illness (PECI)

The PECI is a 25-item instrument designed to measure parental distress related to caring for a chronically ill child [12]. It consists of four subscales that were factor-derived: Guilt and Worry (e.g., I worry that my child’s illness will worsen/return), Emotional Resources (e.g., I feel ready to face challenges related to my child’s well-being in the future), Unresolved Sorrow and Anger (e.g., I am jealous of parents who have healthy children.), and Long-Term Uncertainty (e.g., My hopes and dreams for my child’s future are uncertain.). A pediatrician (first author) and a psychologist translated the PECI questions into Japanese. These questions were then back-translated by language experts at a translation company. We sent the back-translated questions to the author of the original scale and received permission to use the translated PECI questions. A pediatrician and a child psychiatrist (second author) confirmed the validity of the questions by evaluating the consistency between the Japanese text of the questions and the complaints of Japanese parents of the children with cancer. Internal consistency values for the Guilt and Worry, Unresolved Sorrow and Anger, Long-Term Uncertainty, and Emotional Resources factors in this study were 0.80, 0.58, 0.68, and 0.65, respectively.

#### 2.2.4. Parent-Child Communication about the Disease

A parent rated the parent-child communication as “0” if they did not inform the child of the disease at all, “1” if they informed the child that he/she had a disease that must be treated without using the word “cancer” or relaying the possibility of recurrence, and “2” if they fully informed the child of the disease including the diagnosis and prognosis of cancer.

#### 2.2.5. Disease and Treatment Characteristics

Intensity of the treatment the child received was determined by use of Intensity Treatment Rating 3.0 (ITR-3.0) [29]. ITR-3.0 classifies the cancer treatment the child received to four levels, as follows: Level 1, least intensive treatment (e.g., surgery only for all tumor types except brain tumors); Level 2, moderately intensive treatment (e.g., chemotherapy for low, standard, or intermediate risk acute lymphoblastic leukemia); Level 3, very intensive treatment (e.g., chemotherapy for high risk, very high risk, or T-cell acute lymphoblastic leukemia); Level 4, most intensive treatment (e.g., hematopoietic stem cell transplantation).

We determined whether each child had a complication from cancer or a treatment by using the judgment of the attending physician as reported in the inpatient and outpatient records. We referred to the children’s and parents’ complaints, laboratory data and imaging study results. The child’s complication from cancer or treatment at enrollment was denoted as “0” if the child had no complication at enrollment, “1” if the child had a complication at enrollment without disability, and “2” if the child had a complication with disability. For example, if a child had no symptoms but had an imaging abnormality, the child was assigned a value of “1”.

### 2.3. Statistical Analyses

Pearson’s correlation coefficients were calculated to assess inter-correlations between continuous variables such as age, duration of admission, the CBCL total problems *T* score, each subscale of the PECI, and IES-R total scores. Spearman’s correlation coefficients were calculated to assess inter-correlations of categorical variables or ordinal variables, such as the child’s sex, parental educational level, and parent-child communication about disease, with those variables or with continuous variables. Forced entry multiple regression analyses were performed for analyzing the multivariate models. CBLB total problems in the enrolled children and Long-term Uncertainty (a PECI subscale) in their parents were used as dependent variables. Variables significant at a bivariate level were included in the regression equations. Adjusted *p*-values of multiple regression analyses were calculated using Hommel’s procedure [30]. All analyses were conducted with the SPSS software version 23.0 (SPSS, Chicago, IL, USA) for Windows and the R version 3.1.1 (R Foundation for Statistical Computing, Vienna, Austria).

### 2.4. Ethics Statement

This study was conducted in accordance with the Declaration of Helsinki, and approval for this study was obtained from the ethics committee of the University of Tsukuba on 16 February 2008 (the project identification code: H19-216). Participants (33 mothers and one father) were informed, both orally and in written form, of the purpose of this study, the guaranteed protection of privacy, and the right to refuse to participate. Written consent was then obtained from the parents.

## 3. Results

### 3.1. Demographic Characteristics

A total of 39 parents (38 mothers and one father) from 39 families were approached, and 37 parents (36 mothers and one father) from 37 families agreed to participate. A total of 34 participating parents were included in the final analyses: three parents were excluded because of missing data. The data regarding the children of the participating parents were obtained from the parents and medical records.

Table 1 presents characteristics of the final parent-child pairs. The parental age ranged from 22 to 52 years (mean 37.9, SD 6.4). Of the 34 children, 17 (50.0%) were girls and all 34 (100%) were Japanese. Their ages at enrollment ranged from four to 17 years (mean, 10.5; SD 3.9), and those at diagnosis ranged from zero to 16 years (mean, 6.4; SD 4.3). Cancer diagnoses were as follows: acute leukemia, 19 (55.9%); lymphoma, six (17.6%); brain tumor, four (11.8%); and other solid tumor, five (14.7%).

### 3.2. Proportions of Clinically Relevant Behavior Problems and Parental Post-Traumatic Stress Symptoms (PTSS)

Table 2 presents means, standard deviations, and ranges of CBCL, IES-R, and PECI scores in this study. The mean CBCL total problem *T* score in the present study was 55.4 ± 13.8. Using 63/64 as the CBCL *T* score cutoff, nine (26.5%) had clinically relevant total problems, seven (20.6%) had clinically relevant internalizing problems, and eight (23.5%) had clinically relevant externalizing problems. The mean IES-R total score for the parents in the present study was 15.3 ± 12.6. Using 24/25 as the IES-R cutoff, PTSS were present in eight of the 34 parents (23.5%).

### 3.3. Associations between Children’s Behavioral Problems and Their Parents’ Psychosocial Problems

Table 3 presents correlations of demographic, medical, and psychosocial factors of the children with cancer and their parents. The CBCL total problems *T* score of the children with cancer correlated significantly and positively with the total IES-R scores (*r* = 0.596, *p* < 0.001).

### 3.4. Factors Correlated with Psychosocial Problems of the Child with Cancer

The number of close friends of the child correlated significantly and negatively with the CBCL total problems *T* score (*r* = −0.382, *p* = 0.026). Demographic and medical factors, such as the child’s age, and ITR-3.0, did not correlate significantly with the CBCL total problems *T* score (Table 3).

### 3.5. Factors Correlated with the Psychosocial Problems of the Parents

Parental IES-R total scores were significantly positively correlated with ITR-3.0 (ρ = 0.425, *p* = 0.008) as well as parental Guilt and Worry (PECI subscale) (*r* = 0.521, *p* < 0.001). The number of close friends of the child was significantly negatively correlated with the parental IES-R total scores (*r* = −0.400, *p* = 0.008). Additionally, parents of boys with cancer had fewer PTSS than did those of girls (ρ = −0.441, *p* = 0.009).

Parental Long-term Uncertainty (a PECI subscale) was significantly positively correlated with ITR-3.0 (ρ = 0.600, *p* < 0.001), and was negatively correlated with the child’s age at diagnosis (*r* = −0.356, *p* = 0.024) and parental Emotional Resources (*r* = −0.372, *p* = 0.030). Parents who informed their child of the disease in more detail had significantly lower Long-term Uncertainty scores than did those who provided fewer details to their children (ρ = −0.397, *p* = 0.022) (Table 3).

Parental Guilt and Worry (a PECI subscale) was significantly positively correlated with the duration of hospitalization (*r* = 0.401, *p* = 0.019), and was significantly negatively correlated with the number of close friends the child had (ρ = −0.362, *p* = 0.035).

Unresolved Sorrow and Anger (a PECI subscale) was significantly positively correlated with the duration of hospitalization (*r* = 0.387, *p* = 0.024) (Table 3).

The bivariate correlations among all the independent variables entered in multiple regression analyses are presented in Table 3 and Table 4.

### 3.6. Multiple Regression Analyses with Forced Entry

The CBCL total problems *T* score was correlated with the IES-R total scores and the number of close friends of the child in bivariate analyses, as shown in Table 3. However, in multiple regression analysis, only IES-R total scores continued to independently predict the variance of the CBCL total problems score at a significant level (Table 5).

ITR-3.0 independently predicted the variance of parental Long-term Uncertainty after adjustment for the child’s age at diagnosis, although the latter was correlated with parental-child communication about the disease in bivariate analysis (Table 6).

## 4. Discussion

Our study indicated that approximately one-quarter of the children with cancer had clinical general behavioral problems: internalizing problems such as anxiety, depression, or social withdrawal; or externalizing problems such as antisocial and aggressive behavior. The mean CBCL total problems *T* score in the present study was similar to that reported by Barrera et al. for children diagnosed within the past three months (52.5 ± 10.9) and to that reported in a study of children diagnosed with cancer, excluding brain tumor, within the past four months (49.5 ± 12.0), all within the clinically relevant range [5,31]. We found that emotional and behavioral difficulties in some children with cancer persist for a long time after treatment.

Approximately one-quarter of parents of a child with cancer had PTSS. The proportion of parents having PTSS in the present study was similar to that of a previous study in Japan reported by Ozono (20.7% of mothers, 22.6% of fathers) [32], although it was lower than that in a childhood brain tumor survivor study reported by Bruce et al. (29%) [33]. The mean IES-R score for parents in the present study was also similar to that reported by Ozono (mothers: 15.0 ± 12.4, fathers: 16.0 ± 14.3), although it was lower than those reported in studies including patients in active treatment by Phipps et al. (mothers: 21.9 ± 17.9) and Kazak et al. (mothers: 43.6 ± 14.0, fathers: 32.6 ± 21.5), and in a childhood brain tumor survivor study reported by Bruce et al. (25.2 ± 20.75) [32,33,34,35]. Parental PTSS seems to decrease after treatment, but some parents continued to experience PTSS.

The present study included children aged as young as four to six years, whereas the Ozono study in Japan did not [32]. In Japan, studies of emotional and behavioral functioning in preschool children with cancer and their parents have been rare, although a considerable number of children with cancer are preschoolers at diagnosis. More extensive evaluation of emotional and behavioral functioning in preschoolers with cancer and their parents is needed.

In our sample, the emotional and behavioral problems of children with cancer were associated with parental PTSS, but not by demographic or medical factors. To our knowledge, this is the first report of a study in Japan that revealed the relationships between the emotional and behavioral difficulties of children with cancer and parental PTSS, even after excluding the effects of potential confounders by multivariate analysis. There are few reports about psychosocial functioning of children with cancer and their parents in Japan compared to those in western countries. Particularly, very few reports have referred to the relationship between the psychosocial functioning of children with cancer and that of their parents, although Ozono has reported that PTSS in a child with cancer was correlated with that in his/her mother by bivariate analysis [32]. Much previous research carried out in western countries revealed the correlation between the psychosocial functioning of children with cancer and that of their parents. Some previous research demonstrated that self-reported PTSS in the child with cancer and PTSS in their parents were significantly correlated [35,36]. Other studies also found that PTSS in both parents of childhood cancer survivors was associated with PTSS in those individuals [37]. According to the theory of attachment, parents play a role as a secure base when their children face a traumatic event [38]. Parents who suffer from PTSS are thought to have difficulty assuming this role [39]. A previous longitudinal study, which revealed that initially high PTSS in mothers was related to poorer recovery from PTSS in their children, supports this suggestion [40]. In order to improve emotional and behavioral difficulties of children with cancer, we must evaluate parental vulnerability to the development of PTSS and provide adequate intervention as needed.

The parents of children who received more intensive treatment had higher long-term uncertainty. Previous studies that excluded children with relatively severe cancer, such as brain tumor, failed to establish an association between objective medical factors and psychosocial functioning in either the children or their parents [16,17]. Recently, some studies that included patients with relatively severe cancer revealed that severely intensive treatment and late effects of treatment were associated with poor psychosocial outcomes [41]. Therefore, we hypothesized that either the children who received more intensive treatment or their parents would develop more psychosocial difficulties. Our results indicated that these parents developed more psychosocial difficulties, whereas the children did not. These findings suggest that parents are at greater risk of being distressed by the experience of their children’s cancer than the children themselves. More studies that include children with relatively severe cancer are needed.

While the number of close friends was found to be significantly correlated with parental PTSS and emotional and behavioral functioning of the children by bivariate analyses, it did not exert a significant independent effect on the variance in the emotional and behavioral functioning of the children in the regression models. Friends are one of the most important social support resources for children [15]. In addition, the number of close friends the child had may reflect the amount of social support of the parents as well, because parents often communicate with family members of the child’s close friends. Although social support is an important factor in preventing PTSS development after a traumatic event, previous studies on children with cancer did not conclude that social support improved the psychosocial functioning of the children or their parents [15]. Our study suggested the possibility that the children’s close friends may support not only the children with cancer but also their parents. Children with cancer must be hospitalized for a long time. We may support these children and their parents by encouraging them to make an effort to continue to connect with their friends even during hospitalization. In addition, creating peer groups of children with cancer and their families may prevent development of PTSS.

By bivariate analysis, parents who informed their child of the disease had lower long-term uncertainty, but this was not significant in multivariate analysis. In both bivariate and multivariate analysis, there was no significant association between psychosocial functioning of the children with cancer and parent-child communication about the disease. We demonstrated that the child’s knowledge of their disease does not increase the parental burden or the child’s psychosocial difficulties, although in a 2003 survey, 43.5% of Japan’s pediatric oncologists thought the child’s knowledge of his/her cancer diagnosis would increase the parental burden [19]. Our findings should encourage parents and physicians to try to inform children with cancer of their disease. Physicians must continue to support parent-child communication about the disease for a long time after treatment.

This study had several limitations. First, it was a one-time, cross-sectional survey, which cannot establish causal directionality. The second limitation is that we could not use randomization to categorize parents according to whether they informed the child of the disease. Parents who did not inform may have had psychosocial difficulties before the cancer diagnosis. The third limitation of this study is its small sample size. Establishing significant relationships between psychosocial functioning in the children and the various factors analyzed may have been impossible because the study was underpowered. The fourth limitation is that parental PTSS and CBCL were both reported by the parents of the child with cancer. We cannot deny the possibility that parents with more severe PTSS are more likely to report that their children have greater problems.

## 5. Conclusions 

A quarter of the children with cancer enrolled in this study had psychosocial problems. In Japan, psychosocial problems in children with cancer are related to parental PTSS, not demographic and medical factors. Our take-home message is that healthcare workers should evaluate the vulnerability to PTSS of parents of a child with cancer and support the parents.

## Figures and Tables

**Table 1:** Demographics and illness factors of enrolled children with cancer and their parents.

Children	*n* = 34	Parents	*n* = 34
Sex, *n* (%)		Sex, *n* (%)	
Girl	17 (50.0)	Mother	33 (97.1)
Boy	17 (50.0)	Father	1 (2.9)
Age at diagnosis, M ± SD (years)	6.4 ± 4.3		
Age at enrollment, M ± SD (years)	10.5 ± 3.9	Age at participation,	37.9 ± 6.4
Years post-treatment, M ± SD (years)	4.3 ± 2.6	M ± SD (years)	
Duration of hospitalization, M ± SD (months)	10.9 ± 6.1		
ITR-3.0, *n* (%)		Educational level, *n* (%)	
Level 1–2	15 (44.1)	High school or less	18 (52.9)
Level 3–4	19 (55.9)	More than high school	15 (44.1)
Complication at enrollment, *n* (%)		No answer	1 (2.9)
Present	11 (32.3)		
None	23 (67.6)		

ITR: Intensity of Treatment Rating.

**Table 2:** Mean Child Behavior Checklist (CBCL), Impact of Event Scale-Revised (IES-R), and Parent Experience of Child Illness (PECI) scores.

Variables	Mean	SD	Range (Min–Max)
**Children**			
CBCL *T* score			
Total problems (33–100)	55.4	13.8	33–90
Internalizing problems (42–100)	56.5	10.8	42–82
Externalizing problems (40–100)	55.2	12.2	40–90
**Parents**			
IES-R			
Total scores (0–88)	15.3	12.6	0–44
Intrusion (0–32)	5.8	4.1	0–16
Avoidance (0–32)	4.9	5.1	0–16
Hyperarousal (0–24)	4.5	4.9	0–15
PECI			
Long-term Uncertainty (0–4)	1.0	0.8	0.0–3.5
Guilt and Worry (0–4)	1.5	0.7	0.5–3.4
Unresolved Sorrow and Anger (0–4)	0.4	0.6	0.0–2.0
Emotional Resources (0–4)	3.1	0.8	0.6–4.0

**Table 3:** Correlations of demographic, medical, and psychosocial factors of the children with cancer and their parents.

Variables	Psychosocial Problems of the Children	Psychosocial Problems of the Parents				
	CBCL	IES-R	PECI			
	Total Problems *T* Score	Total Scores	Long-Term Uncertainty	Guilt and Worry	Unresolved Sorrow and Anger	Emotional Resources
**Children**						
Sex	−0.204	−0.441 **	−0.175	−0.093	−0.260	0.121
Age at diagnosis	−0.162	−0.289	−0.356 *	−0.135	−0.048	0.039
Age at enrollment	−0.325	−0.201	−0.241	−0.252	−0.030	0.211
Duration of hospitalization	0.012	0.152	0.090	0.401 *	0.387 *	0.233
Years post-treatment	0.010	0.232	0.216	−0.050	0.056	0.263
Complication at enrollment	0.130	0.168	0.179	0.311	0.006	−0.086
ITR-3.0	−0.046	0.425 **	0.600 **	0.154	0.108	−0.134
Number of close friends of the child	−0.382 *	−0.400 **	−0.328	−0.362 *	−0.430	−0.019
CBCL total problems *T* score	−	0.596 **	0.097	0.283	−0.003	−0.172
**Parents**						
Age at participation	−0.325	−0.265	−0.247	−0.116	0.105	0.167
Education level	−0.154	−0.158	−0.109	−0.281	−0.143	0.093
Parent-child communication about the disease	−0.161	−0.063	−0.397 *	−0.080	−0.205	0.270
IES-R total scores	0.596 **	−	0.286 *	0.521 **	0.308	−0.253
**PECI**						
Long-term Uncertainty	0.097	0.286	−	0.286	0.245	−0.372 *
Guilt and Worry	0.283	0.521 **	0.327	−	0.202	−0.101
Unresolved Sorrow and Anger	−0.003	0.308	0.245	0.202	−	−0.093
Emotional Resources	−0.172	−0.253	−0.372 *	−0.101	−0.093	−

CBCL: Child Behavior Checklist, IES-R: Impact of Event Scale-Revised, PECI: Parent Experience of Chronic Illness, ITR: Intensity of Treatment Rating, * *p* < 0.05, ** *p* < 0.01.

**Table 4:** Correlations of variables for multiple regression analyses.

Variables	1.	2.
1. Child’s age at diagnosis	–	–
2. ITR-3.0	−0.060	–
3. Parent-child communication about disease	0.508 **	−0.099

ITR: Intensity of Treatment Rating ** *p* < 0.01.

**Table 5:** Factors related to CBCL total problems by multiple regression analysis.

Predictor Variables	β	*SE*	*p*	Adjusted *p*
Parental IES-R total score	0.529	0.183	0.003	0.012
Numbers of close friend of the child	−0.130	3.265	0.441	0.441
Adjusted *R*^2^	0.327			

**Table 6:** Factors related to Long-term Uncertainty of the parents by multiple regression analysis.

Predictor Variables	β	*SE*	*p*	Adjusted *p*
Child’s age at diagnosis	−0.173	0.028	0.263	0.441
ITR-3.0	0.518	0.116	0.001	0.005
Parent-child communication about the disease	−0.234	0.150	0.138	0.394
Adjusted *R*^2^	0.423			

ITR: Intensity of Treatment Rating.

## Abbreviations

The following abbreviations are used in this manuscript:

IES-R	Impact of Event Scale-Revised
PECI	Parent Experience of Child Illness
CBCL	Child Behavior Checklist
PTSS	Post-traumatic stress symptoms
PTS	Post-traumatic stress
PTSD	Post-traumatic stress disorder
ITR	Intensity of Treatment Rating Scale
